# Mental distress in the general population in Zambia: Impact of HIV and social factors

**DOI:** 10.1186/1471-2458-9-298

**Published:** 2009-08-18

**Authors:** Peter J Chipimo, Knut Fylkesnes

**Affiliations:** 1 University of Zambia, School of Medicine, Department of Community Medicine, P/Bag Rw X1, Lusaka, Zambia; 2 University of Bergen, Faculty of Medicine, Centre for International Health, PO Box 7804, 5020, Bergen, Norway

## Abstract

**Background:**

Population level data on mental health from Africa are limited, but available data indicate mental problems to represent a substantial public health problem. The negative impact of HIV on mental health suggests that this could particularly be the case in high prevalence populations. We examined the prevalence of mental distress, distribution patterns and the ways HIV might influence mental health among men and women in a general population.

**Methods:**

The relationship between HIV infection and mental distress was explored using a sample of 4466 participants in a population-based HIV survey conducted in selected rural and urban communities in Zambia in 2003. The Self-reporting questionnaire-10 (SRQ-10) was used to assess global mental distress. Weights were assigned to the SRQ-10 responses based on DSM IV criteria for depression and a cut off point set at 7/20 for probable cases of mental distress. A structural equation modeling (SEM) was established to assess the structural relationship between HIV infection and mental distress in the model, with maximum likelihood ratio as the method of estimation.

**Results:**

The HIV prevalence was 13.6% vs. 18% in the rural and urban populations, respectively. The prevalence of mental distress was substantially higher among women than men and among groups with low educational attainment vs. high. The results of the SEM showed a close fit with the data. The final model revealed that self-rated health and self perceived HIV risk and worry of being HIV infected were important mediators between underlying factors, HIV infection and mental distress. The effect of HIV infection on mental distress was both direct and indirect, but was particularly strong through the indirect effects of health ratings and self perceived risk and worry of HIV infection.

**Conclusion:**

These findings suggest a strong effect of HIV infection on mental distress. In this population where few knew their HIV status, this effect was mediated through self-perceptions of health status, found to capture changes in health perceptions related to HIV, and self-perceived risk and worry of actually being HIV infected.

## Background

Mental disorders make a substantial independent contribution to the burden of disease worldwide. It is estimated that, neuropsychiatric conditions account for up to 15% of all disability-adjusted life-years, and up to 30% of those attributable to non-communicable diseases. Neuropsychiatric disorders also account for 1.2 million deaths every year. [1,2] These figures are most likely underestimated as official statistics in low and middle income countries are scanty and unreliable. [2] In sub-Saharan Africa, it has been reported that 20–30% of primary health care centre attendees present with depressive symptoms as the first or secondary reason for seeking medical care.[3] A study conducted in Tanzania revealed a 41.6% prevalence of depressive symptoms among primary health care patients while a similar study in Uganda reported a 20–30% prevalence of psychological disorders and depression among health care seekers.[4,5]. These research findings have also shown heightened risk for common mental disorders among the women i.e. a female to male ratio of 1.5–2.0. Other determinants have been found to include low socioeconomic position indicated by poor access to resources, unemployment and low educational attainment. It has also been shown to be higher among those with poor socio-support networks such as the unmarried, widowed and divorced. [6-8]

Mental disorders interact with many other health conditions, thus predicting the onset and progression of both physical and social disability. Several studies have established independent associations between mental disorders and an excess in all-cause mortality risk. In a meta-analysis, Saz and Dewey found pooled odds ratio of 1.7 for a diagnosis of depression and subsequent all-cause mortality. [9] Of particular relevance for this investigation is the interaction between mental disorders and HIV infection. Evidence has shown a heightened risk for contracting HIV infection among those with mental disorders. [10,11] Socioeconomic, psychological and biological factors [1,12-14] have been reported as predisposing factors in HIV infection and have also been found to be relevant factors in mental distress related HIV disease progression. [1,12]. Mental disorders can also mediate delayed help seeking, diagnosis, poor compliance to medication [15] and can predict drop out from HIV-risk reduction programmes. [16,17]

Although evidence from low income countries is limited, fairly consistent associations have been reported between HIV and poor mental health with most published studies showing differing but high percentages of mental distress, e.g. observations from South Africa with prevalence of 40% contrasting a study in rural Ethiopia showing14%.[18,19] A meta-analysis of studies comparing HIV positive and HIV negative groups revealed a significant difference in the prevalence of major depression (HIV positive 9.4% vs. HIV negative 5.2%, OR 2.0, CI 1.3–3.0). [20] These studies have indicated that mental distress can be prevented by increasing the awareness about it among mental health personnel. [10,11] The benefit of screening for mental distress is especially important among the HIV infected. Left undiagnosed mental distress leads to failure of the HIV positive to deal with their sero-status [19] with implications of increased substance abuse and suicides. All these put together build the case for early identification of patients with mental distress and prompt psychotherapy [21].

Despite the known benefits, the practice of screening for mental distress as it relates to HIV infection is still low in many countries. In Zambia, little is known about the extent of mental distress. [22] Considering that Zambia has a high prevalence of HIV [14,23,24] and assuming that HIV infection negatively affects mental health, this would suggest that the mental distress problem is substantial. However, few population based studies, i.e. covering men and women in the general population, have been conducted in sub-Saharan Africa to examine the relationship between HIV and mental distress. The aim of this study was to investigate the magnitude and determinants of mental distress with particular emphasis on the mechanisms involved in the way HIV infection impacts mental distress by establishing a linear structural equation model.

## Methods

### Measuring mental distress

A wide variety of questionnaires and instruments have been developed over the years to estimate psychological distress in the population, identify high risk groups for mental disorders and monitor the changes over time. The Self reporting questionnaire (SRQ) is an example of such a scale developed as part of a collaborative study on strategies for extending mental health care co-ordinated by the WHO. [11] Although primarily intended for use in epidemiological studies of mental disorders, it is also being used extensively for clinical and other research purposes. [11] It has been judged to be acceptable for most subjects and was found to be appropriate for use in different kinds of settings and countries. [11,19,25] It is now a well established responder-reported questionnaire for measuring psychological distress or the degree of global mental distress [11]. In this study we use a Self reporting questionnaire with 10 symptom questions which are scored on a dichotomous scale but do not probe to evaluate symptom severity [11]. It has also been shown that the shorter SRQ-10 performs just as well as the longer scales for evaluating mental distress, for example SCL-25 and SF-36 as well as other shorter scales such as SCL-10, SCL-5 and MHI-5. It has been suggested that it can be used in the place of longer scales for evaluating mental distress and yield comparable results. [25]

### The model

In this model, a set of several linear equations are connected in a system. Our central theoretical premise is that mental distress has biological, social and psychological determinants. Therefore four hypotheses have been developed and require further testing. Firstly, we propose that, demographic characteristics and socioeconomic position indicators are directly and indirectly associated with mental distress. [19] Male gender, young age, (15–25 years), educational attainment, social support networks (being married vs. single) and wealth index are found to be positively associated with better mental health status. [26] Secondly, self-rated health, and self HIV risk perception and worries of being infected (HIV risk and worry) are also associated with better mental health. [27] Thirdly, we propose that HIV infection has both direct and indirect effects on mental distress. [27] Direct, mediated by biological factors and indirectly mediated through self-rated health and HIV risk and worry of being infected. [27,28]

### Population and Sampling Procedures

The population-based HIV surveys have been conducted in Zambia every third year since 1996 in selected rural (Kapiri Mposhi) and urban (Chelston) communities. For this investigation we used data from the survey conducted in 2003 (n = 4466) using stratified random-cluster sampling method. The detailed methods of the surveys conducted have been reported else where. [23,29] The sampling frame consisted of 24 clusters (Standard Enumeration Areas) in Chelston and 26 clusters in Kapiri Mposhi. The cluster defined the primary sampling unit of the study. Using probability proportional to size, 10 clusters were selected from each of the areas. All household members 15–59 years in the selected clusters were listed and invited to participate in the study

### Data Collection

#### Personal Interviews

The data was collected at household level by trained enumerators. Personal interviews were carried out with all eligible and willing household members in order to collect information on socio-demographic characteristics, health seeking behaviour, sexual behaviour and perception regarding HIV. Details of data collection methods have been described elsewhere. [23]

Information on mental distress was collected using Self-reporting questionnaire-10 (SRQ-10) which is a 10 item questionnaire containing basically two domains namely, depressive symptoms and somatisation. The SRQ-10 is based on a dichotomous response answer system to the questions given in table 1 "In the past 30 day*s*".... It is apparent from the review of studies done using the SRQ that no global or generally applicable cut-off score can be recommended and that each study should determine its own cut-off point. [11,25] The rationale for setting the cutoff point of >7/20 in this study was based on the DSM-IV classification. Firstly each symptom was weighted according to severity with the more severe symptoms getting higher ranking, while the less severe symptoms got lower ranking (table 1). The cutoff point was then based on the DSM-IV requirement of 5 or more symptoms under the headings; thoughts of suicide, loss of interest or pleasure and depressed mood. These raw weights are then summed up in a transformed summative index ranging from 1–20. This continuous mental distress variable was used in the SEM model. Based on the DSM-1V criteria for depression which requires 5 or more items of the above that would represent a change in previous functioning, or at least either a depressed mood or loss of interest or pleasure, a cut off point of ≥7 for mental distress was set. [30]

**Table 1:** SRQ-10 diagnostic symptoms and weights

**Diagnostic Symptom**	**Question**	**Weight**
A. Thoughts of Death	*Has the thought of ending your life been on your mind?*	5
B. Loss of interest or pleasure	*Is your daily life suffering?*	3
	*Are you unable to play a useful part in your life?*	3
	*Do you find it difficult to enjoy your daily activities?*	3
C. Depressed mood	*Do you sleep badly?*	1
	*Do you cry more than usual?*	1
	*Do you have difficulties deciding?*	1
	*Are you tired all the time?*	1
	*Do you often have Headaches?*	1
	*Is your digestion poor?*	1

#### Laboratory Investigation

At the end of the interview the participants were requested to provide a saliva sample for HIV testing. The saliva samples were collected on an anonymous linked HIV testing protocol. BIONOR HIV 1&2 (BIONOR AS, Skein, Norway) paramagnetic particle assay was used as the first line test. The reactive samples were subsequently tested again using rapid test (Capillus HIV-1/HIV-2, Cambridge Biotechnology). Samples with discrepant results were sent for a confirmatory Western blot. [29]

### Statistical Analysis

Data was analysed using SPSS version 15.0 and cluster effect accounted for in the analyses. Characteristics of the study population in terms of demographic, socioeconomic and HIV status were described using descriptive statistics. These were compared by sex, residence and HIV status in cross-tabulations. Only respondents with valid HIV results and aged 15–49 years were included in the subsequent analysis (N = 4466).

Analysis of Moment Structures (AMOS) version 7.0 was used in the Structural Equation Modelling (SEM) [31] to confirm the theoretical built model that included the underlying factors (demographic and socioeconomic), intermediate factors (self-rated health and HIV risk and worry), HIV status, knowledge of own HIV status and consequently mental distress. Firstly the model was designed and fitted based on the hypotheses. Secondly, regression coefficients and their significant levels for each parameter were calculated. Thirdly, relative chi-square statistic, goodness of fit index (GFI), [32] adjusted goodness of fit index (AGFI), [32] comparative fit index (CFI) [33] and root mean square error of Approximation (RMSEA) [33] index model fitness were obtained for model diagnostics. The criteria used were chi-square statistic of more than 0.50, GFI of equal or greater than 0.95, AGFI of equal or greater than 0.90, CFI greater or equal to 0.90 and RMSEA of less or equal to 0.08. [31-33] Addition of correlations between error terms, considering only the significant correlations as well as putting constraints on the parameters was done to improve the model. The total direct and indirect effects of the underlying and intermediate factors were calculated using standardised regression weights of each pathway with the maximum likelihood ratio as the method of estimation.

In the model, marital status was dichotomised to ever married variable grouped as, single (single, engaged, Living as married) vs. married (Married, divorced, separated, widowed). Level of education was used as a continuous variable (number of years in school). A wealth index scale was constructed using factor analysis from six questions assessing wealth status. A second summative index (HIV risk and worry) was constructed which combined responses to the questions concerning self perceived risk of HIV infection (In your situation, do you think that you are risk of getting (catching) HIV? 1 = you are not at risk, 2 = the risk is moderate or 3 = the risk is high or 4 = the risk is very high) and worry about being HIV infected (How worried are you about actually being infected by HIV/AIDS? 1 = Always worried, 2 = Sometimes worried, or 3 = Seldom worried, or 4 = Never worried). Self-rated health was also used as a continuous variable. (How would you say your health is at the moment? Is it 1 = Excellent, 2 = Good, 3 = Fair, 4 = Poor, 5 = Very poor). The dependant variable mental distress was also used as a continuous variable with scores ranging from 1–20. There was insignificant evidence of interaction between the variables and so no interaction terms were included. Measures were also done to account for design effect which had the effect of widening confidence interval.

### Ethical Clearance

The survey received ethical clearance from the University of Zambia Ethics Committee. Additionally, participation in the survey was based on written informed consent. Participants were counselled and informed that the information obtained was purely anonymous and for research purposes. Participants interested in knowing their HIV status were offered voluntary counselling and testing at home.

## Results

### Characteristics of study population and extent of mental distress

Table 2 shows a pattern observed from an item to item analysis of the symptoms of mental distress. Daily life suffering (27.4%), frequent headaches (27.4%) and difficulty enjoying life (23.6%) were the most common symptoms among the HIV positive rural males. Comparatively, urban males complained more of poor sleep (21.4%), difficulty deciding (18.3%) and daily life suffering (18.3%). Among the HIV infected rural females poor sleep (23.6), daily life suffering (21.6) and frequent headaches (21.6) were the most common complaints. The urban female population presented with difficulty deciding (33.9%), frequent headaches (28.4%) and difficulty enjoying life (24.0%). Thoughts of suicide represented less than 6% of the total study population. Among the HIV infected, women (8.9%) reported contemplating suicide more than their male (3.0%) counterparts (p = 0.003). A similar pattern was noted among the HIV uninfected (men 3.6%, women 6.4%, p = 0.001)

**Table 2:** Observed Symptoms of Mental Distress by Sex, Residence and HIV status

	**Rural**								**Urban**							
	
	Male (n = 818)				Female (n = 1050)				Male (n = 1042)				Female (n = 1540)			
	
**Symptom**		neg%	pos%			neg%	pos%			neg%	pos%			neg%	pos%	
	Total%	n = 712	n = 106	p	Total%	n = 902	n = 148	p	Total%	n = 911	n = 131	p	Total%	n = 1206	n = 334	P
Sleep badly	13.6	12.8	17.9	0.15	15.7	14.4	23.6	0.00	11.5	10.1	21.4	0.00	16.2	14.6	21.9	0.00
Cry more	3.1	2.9	3.8	0.65	8.4	7.8	12.8	0.04	3.4	3.2	4.6	0.41	10.7	10.0	13.5	0.06
Difficulty enjoying life	15.8	14.7	23.6	0.02	14.4	13.7	18.9	0.10	19.4	19.7	17.6	0.57	18.9	17.4	24.0	0.01
Difficulty deciding	20.2	20.2	20.8	0.90	14.5	14.2	16.2	0.52	23.1	23.8	18.3	0.16	27.3	25.5	33.9	0.00
Daily life suffering	20.0	19.0	27.4	0.04	18.3	17.7	21.6	0.26	14.0	13.4	18.3	0.13	15.0	12.8	22.8	0.00
Unable to play useful part in life	11.4	11.2	13.2	0.55	10.7	10.2	13.5	0.23	9.9	9.1	15.3	0.03	10.3	9.6	12.9	0.08
Thoughts of suicide	2.0	2.2	0.9	0.38	5.1	4.5	8.1	0.07	5.2	5.3	4.6	0.74	8.1	7.8	9.3	0.38
Tired all the time	13.3	12.4	19.8	0.04	12.0	11.0	18.2	0.01	7.7	6.8	13.7	0.01	11.8	10.8	15.6	0.02
Headache often	18.2	17.0	27.4	0.01	23.8	24.1	21.6	0.51	17.3	17.4	16.8	0.87	27.6	27.3	28.4	0.69
Poor Digestion	14.3	13.1	22.9	0.01	8.6	8.2	10.8	0.30	7.0	6.7	9.2	0.30	6.2	5.3	9.3	0.01
Mental distress*	13.6	12.9	18.1	0.15	15.4	14.3	21.6	0.02	11.5	11.1	13.7	0.38	15.4	13.0	24.1	0.00
Positve Negative ratio:		1	1.40			1	1.51			1	1.23			1	1.85	

* Mental distress cut off 7/20 questions given weights and arbitrary cut off point set from literature based on the DSM IV criterion for depression

The prevalence of HIV was 13.6% in rural and 18.0% in urban areas (Table 2). Knowledge of own HIV results was reported by 13.6%, and this knowledge differed clearly by residence, 8.3%in rural and 17.4% in urban (p < 0.001). Of these, 43.4% lived in rural areas and 56.6% were urban residents. The mean (SD) age of the men was 27(8.8) years and 27(8.9) years for women. Marital status differed substantially by residence, i.e. proportion being married was 66.7% in the rural and 81.4% in the urban population. Whereas 64.3% of the urban residence had attained at least 10 years of education, the respective proportion was 15.4% among rural residents.

The prevalence of mental distress in men was 12.4% and15.4% in women (*χ*^2 ^= 8.033, DF = 1, p = 0.005, i.e. a prevalence ratio, women: men of 1.24. This ratio was highest in the age-group 15–24 years of 1.6). Mental distress did not differ by residence (*χ*^2 ^= 0.190, DF = 1, p = 0.663) and only tended to increase by age. Mental distress was affected by educational attainment, i.e. the prevalence among urban residents was 2.3 times higher among the group with the lowest vs. the highest level of education, and the respective rural ratio was 1.94. (Table 3) A consistent pattern of higher mental distress among the HIV infected was observed by sex and residence, and the prevalence ratio infected vs. non-infected was 1.61. (*χ*^2 ^= 24.141, DF = 1, p = 0.000)

**Table 3:** Proportion of participants and the prevalence of mental distress by residence and background characteristics

	**Rural**			**Urban**			**Total**		
**Characteristics**	Number	%	Mental distress (%)	Number	%	Mental distress (%)	Number	%	Mental distress (%)

**Age**									
15–19	338	18.0	12.1	480	18.5	11.8	818	18.3	11.9
20–24	362	19.3	13.3	464	17.9	12.3	826	18.5	12.7
25–29	290	15.5	16.2	391	15.1	15.5	681	15.2	15.8
30–39	387	20.6	15.8	614	23.7	15.3	1001	22.4	15.5
40–49	242	12.9	13.9	351	13.6	14.2	593	13.3	14.0
**Sex**									
Male	822	43.8	13.6	1042	40.2	11.5	1864	41.7	12.4
Female	1055	56.2	15.4	1547	59.8	15.4	2602	58.3	15.4
**Number of years in school**									
0–6	867	46.7	16.1	195	7.5	23.8	1062	23.8	17.5
7	406	21.8	14.8	254	9.8	22.8	660	14.9	17.9
8–9	301	16.2	14.0	472	18.3	16.2	773	17.4	15.3
10–11	93	5.0	15.2	410	15.9	11.5	503	11.3	12.2
>12	194	10.4	8.3	1250	48.4	10.2	1444	32.5	9.9
**Ever married**									
Single	538	33.3	13.6	481	18.6	13.9	1019	22.8	13.7
Married	1079	66.7	15.1	2102	81.4	13.8	3181	71.2	14.2
**Wealth index**									
Low	503	79.3	17.4	820	75.5	26.0	1323	76.9	19.1
Medium	101	15.9	15.1	210	19.3	17.3	311	18.1	15.8
High	30	4.7	10.6	56	5.2	12.4	86	5.0	12.1
**HIV**									
Negative	1621	86.4	13.7	2122	82.0	12.2	3707	83.8	12.9
Positive	256	13.6	20.2	467	18.0	21.2	716	16.2	20.8

### Correlation Coefficients

Table 4 shows the Pearson correlation coefficients matrix of the observed variables. Mental distress was correlated to self-rated health (r = 0.22), wealth index (r = 0.07), risk-worry (r = 0.15), HIV status (r = 0.08), age (r = 0.05) and inversely correlated to school years (r = -0.09). Self rated health was inversely correlated to residence (r = -0.14), school years (r = -0.18), marital status (r = -0.30) and directly correlated to wealth index (r = 0.17) and age (r = 0.18). Risk-worry was correlated to self rated health (r = 0.14) and age (r = 0.10, p < 0.05). HIV status was correlated to self rated health (r = 0.15), risk-worry (r = 0.10) and age (r = 0.21).

**Table 4:** Pearson correlation coefficients matrix of the measured variables

	**1.**	**2.**	**3.**	**4.**	**5.**	**6.**	**7.**	**8.**	**9.**	**10.**	**11.**
**Variables**											
**1**. Residence	1										
**2**. Sex	0.04*	1									
**3**. Age	-0.17**	-0.01	1								
**4**. Ever married	0.17**	-0.07**	-0.05**	1							
**5**. School years	0.54**	-0.13**	-0.07**	0.82**	1						
**6**. Wealth index	-0.71**	-0.01	-0.15**	-0.09**	0.57**	1					
**7**. Self rated health	-0.14**	0.06**	0.18**	-0.03**	-0.18**	0.17**	1				
**8**. Risk-worry	-0.02	-0.01	0.10*	0.01	-0.01	0.02	0.14*	1			
**9**. HIV	0.06**	0.08**	0.21*	0.00	0.00	-0.02	0.15**	0.10*	1		
**10**. Knowledge of own	-0.31**	-0.07	0.08*	-0.01	-0.25**	0.24*	0.09*	0.05	0.03	1	
HIV status											
**11**. Mental distress	-0.01	0.04**	0.05**	0.01**	-0.09**	0.07**	0.22**	0.15**	0.08**	0.04	1

* Correlation significant at 0.05

** Correlation significant at 0.01

### Final Model

**Figure 1:**
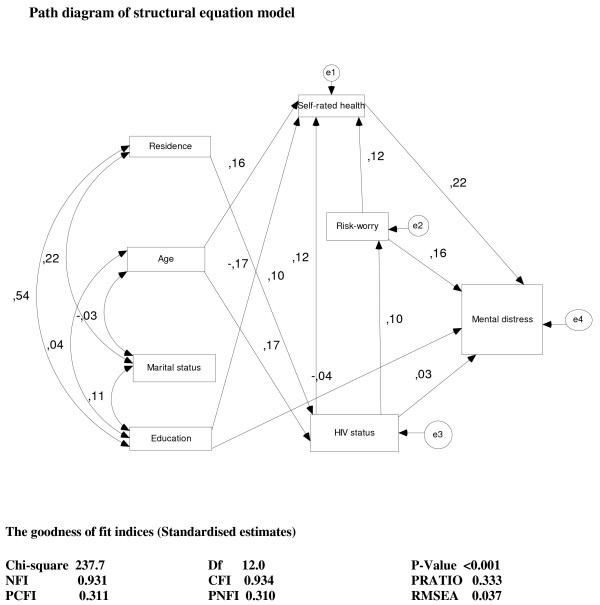
**Significant pathways of the final model and goodness-of-fit indices**. DF = degrees of freedom, NFI = Normed fit index, CFI = comparative fit index, PRatio = Parsimony ratio, PCFI = parsimony comparative fit index, PNFI = parsimony Normed fit index, RMSEA = Root mean square error approximation.

Figure 1 illustrates the final model with significant pathways and their associated goodness of fit indices. The model diagnostics indicated that the underlying factors, residence, school years, ever married and age were inter-correlated. However the error terms of Self-rated health, risk-worry, HIV status and mental distress were not correlated. The observed measures of model fitness were as follows: Chi-square for goodness-of-fit test (*χ*^2 ^= 237.7, DF = 12.0, p < 0.001), baseline comparisons (NFI = 0.931, CFI = 0.934) and parsimony-Adjusted measures (PRatio = 0.333, PCFI = 0.311, PNFI = 0.310, RMSEA = 0.037).

### Structural relationships between observed variables

Table 5 shows significant structural relationships between the underlying, intermediate and outcome variables. Mental distress was directly related to risk-worry (b = 0.16), HIV status (b = 0.03) and self-rated health (b = 0.22). Self-rated health is related to age (b = 0.17), risk-worry (b = 0.12) and HIV status (b = 0.12). It was also directly related to school-years (b = 0.17). Risk-worry is directly related to both HIV (b = 0.09) while HIV status was found to be directly related to residence (b = 0.10) and age (b = 0.10).

**Table 5:** Structural relationships between observed variables

**Observed variables**		**b^b^**	**B^a^**	**P-value^c^**
Age	--->HIV status	0.06	0.10	< 0.001
Residence	--->HIV status	0.07	0.10	< 0.001
HIV	--->Risk-worry	0.47	0.09	< 0.001
HIV	--->Self-rated Health	0.23	0.12	< 0.001
School year	--->Self-rated Health	0.03	0.17	< 0.001
Risk-worry	--->Self-rated Health	0.05	0.12	< 0.001
Age	--->Self-rated Health	0.01	0.17	< 0.001
HIV	--->Mental distress	0.27	0.03	0.04
Self-rated Health	--->Mental distress	1.05	0.22	< 0.001
Risk-worry	--->Mental distress	0.32	0.16	< 0.001
School years	--->Mental distress	-0.04	-0.04	0.002

b^b ^= Unstandardised regression Coefficients

B^a ^= Standardised regression Coefficients

P-value^c ^= P-value for unstandardised regression coefficients

### Mediating Factors

Table 6 shows the total, direct and indirect effects of the observed independent variables on the dependant variables. Self-rated health and risk-worry appear to be important mediators between underlying factors and mental distress. They are also important mediators between HIV status and mental distress. Age is directly related to HIV status (B = 0.17). It is also directly related to Self-rated health (mediated by risk-worry and HIV status, B = 0.17) and indirectly related to mental distress (mediated by risk-worry and HIV status, B = 0.05). Residence is directly related to HIV status (B = 0.10) and indirectly related to mental distress mediated by risk-worry and HIV status with a small total effect B = 0.01). Number of school years is directly related to self-rated health (B = 0.17) and indirectly related to mental distress (mediated by risk-worry and HIV status, B = -0.04). Risk-worry is related to mental distress both directly and indirectly (Total effect B = 0.20). Self-rated health is directly related to mental distress (B = 0.22). HIV status is related to mental distress both directly and indirectly mediated by risk-worry and self-rated health (Total effect = 0.07).

**Table 6:** Total, direct and indirect effects of observed variables

**Observed variables**	**Effect^a^**	**Age**	**Residence**	**School years**	**Risk-worry**	**HIV status**	**Self-rated Health**
HIV Status	Total	0.17	0.10	-	-	-	-
	Direct	0.17	0.10	-	-	-	-
	Indirect	-	-	-	-	-	-
Risk-worry	Total	0.02	0.01	-	-	0.10	-
	Direct	-	-	-	-	0.10	-
	Indirect	0.02	0.01	-	-	-	-
Self-rated health	Total	0.19	-0.01	0.17	0.12	0.13	-
	Direct	0.17	-	0.17	0.12	0.12	-
	Indirect	0.02	0.01	-	-	0.01	-
Mental Distress	Total	0.05	0.01	-0.08	0.20	0.07	0.22
	Direct	-	-	-0.04	0.17	0.03	0.22
	Indirect	0.05	0.01	-0.04	0.03	0.04	-

^a ^Standardised regression weight

## Discussion

We investigated the magnitude and distribution patterns of mental distress and employed a structural equation model to explore mechanisms involved in the impact of HIV on mental distress. Data stem from a population based HIV survey in Zambia using the SRQ-10 as the tool to measure mental distress. The prevalence of HIV was 13.5% and 18.2% for the rural and urban population, respectively, and most of the survey participants (86.4%) did not know their own HIV status. The prevalence of mental distress was somewhat higher among women (15.4%) than among men (12.4%, p = 0.005), but no urban-rural difference was revealed. The results suggest the effect of HIV infection on mental distress to be both direct and indirect, and particularly strong through the indirect effects of poor health ratings and high self perceived risk and worry of HIV infection. In this regard it should be noticed that self-rated health has previously been found to capture changes in health perceptions related to HIV.[28] In the model, this impact appears to be socially patterned with the number of school years being indirectly related to mental distress in a pattern mediated by self-rated health, risk-worry and HIV status. Age and residence were found to be directly related to HIV status but indirectly related to mental distress in a path mediated also by risk-worry and self-rated health.

Although complex, our model obviously represents an oversimplification of the factors at play. The empirical basis for this structural model might be somewhat shaky, but we judge the plausibility of most specifications to be fairly strong. The theoretical basis underlying the specifications of the model is also thought to be fairly strong as the introduced measures in the present model have been judged to cover most of the dimensions postulated by other authors. The fit indices for our model show a close fitting model. However, the chi-square test as a measure of fit is best for models with N = 75 to N = 100. [31,33,34] For N>100, chi square is almost always significant since the magnitude is affected by the sample size, as in our case where N = 4466 (p = 0.000). Chi-square is also affected by the size of correlations in the model: the larger the correlations, the poorer the seeming fit of the model. [34]

The results confirm previous findings suggesting a strong impact of HIV infection on mental distress. [35,36] In this present study only 13% knew their own HIV status, accordingly, we are likely to have measured a combination of HIV-related effects both biological and psychological. Based on HIV epidemiological evidence we can assume that, on a group level, most HIV infections in young people are resent and that in older groups we can expect that the HIV positive on average have been infected longer and thus will have experienced much more serious impact on their immune system. A simple assumption in our analysis will thus be that the difference in mental distress between HIV infected and uninfected will increase by age. The data did not provide clear evidence of this. However, in the suggested path diagram, to model the determinants of mental distress, self-rated health and self perceived risk and worry about being HIV infected (risk-worry) were assumed to capture indirect effects of HIV on mental distress. Self-rated health has previously been found to capture changes in health perceptions related to HIV. [28] Literature on predictors of self-rated health has shown depression as a strong independent determinant even after accounting for physical illness and functional disability. [37,38] Therefore, self-rated health and risk-worry appear to be sensitive indicators of health changes linked to HIV and mental distress. [28,37] We found strong independent associations between HIV and self-rated health, HIV and risk-worry and between self-rated health, risk-worry and mental distress. A possible interpretation is that the three variables are together capturing effects of HIV – being direct as a biological – or an indirect as a psychological effect. These findings need to be followed up by further studies trying to sort out what could be the more biological versus other effects of HIV infection on mental distress. The estimates may also have been biased by measurement errors. We were unable to find comparable studies on mental distress covering the general population of men and women. Most studies found were conducted among selected groups such as homosexuals, injection drug users and hospital/clinic attendees. [18,35,39,40] It was as such difficult to make direct comparisons with other published literature. However, the patterns of association appear to be similar to other published literature. [18,19,41-43]

The SRQ-10 appears to be a practical tool for measuring mental distress although more needs to be done to validate its use in the Zambian context. In order to attain a more accurate standard for the diagnosis of global mental distress, the 10 indicator questions were weighted and a cut off point of 7 set based on the adapted criterion for the DSM-IV classification for depression. [30] The question of generalization of findings (national, regional levels) is difficult to judge. The communities from which this study was conducted were selected on the basis of being reasonably representative in terms of HIV prevalence and cultural mix to the other communities in Zambia. It is likely that the HIV-mental distress relationship can be extrapolated to the national level and to many other countries in the region as well.[29]

Non-participation might have been one of the possible sources of biases in prevalence estimates and associations in this investigation. Whereas refusal to participate was low, the non-participation due to absence was relatively high among men. The 2003 population-based survey was a follow-up of previous surveys in the same populations (since 1995). Previous publications investigating the HIV prevalence trends on the basis of these repeated surveys reported marked HIV declines since 1995, and the authors did not find any sign of substantial bias due to non-response. [29] For the present analysis we are concerned about the extent to which non-response might have biased prevalence of mental distress and the associations. Given an assumption that non-responders were more likely to be mentally distressed than responders, we would have under-estimated the magnitude of distress and most likely reduced the strengths of associations. Men were substantially more likely to be absent, and a possibility given the above scenario is that the difference between men and women was actually under-estimated. However, there were limited opportunities to further assess the magnitude and direction of this type of bias.

There are a number of limitations of this study. One is the cross-sectional nature of the survey data, which limits the validity of statements of causation to statements only about associations. However, our main interest was not to establish causal pathways, but rather patterns of interrelationships in the data that would fit to a better or worse degree the assumptions of our theoretical premise. Strictly speaking just as mental distress can predispose to HIV infection, the converse is also theoretically possible. It should be noted that the associations revealed in cross-sectional data have very often provided reliable indications of actual effects, and the revealed strong indirect association between HIV status and mental distress is in agreement with previous findings [35,40] The ubiquitous problem of omitted variables is also a factor in this present study. The relationship between mental distress and HIV could also be attributable to other factors not included in our analysis. Examples include employment status, other social and economic factors or indeed other stress inducers not included in our analysis. Further, a more optimal design would have been needed to measure the biological effect of HIV infection adequately to include information on HIV clinical staging and CD4 counts. The data were not affected by antiretroviral treatment effects, since such treatment in practical terms was not available in these populations in 2003.

## Conclusion

The results suggest that HIV infection has a substantial effect on mental distress both directly and indirectly. This effect was mediated through self-perceptions of health status, found to capture changes in health perceptions related to HIV, and self-perceived risk and worry of actually being HIV infected. To our knowledge this is the first study to investigate the pattern of relationship between HIV and mental distress by using the structural equation modeling. The use of the structural equation modeling allowed for simultaneous evaluation of the direct and indirect effects of background and intermediate factors on mental distress within the framework of the model. More research is urgently needed into this area in order to understand the epidemiology of mental distress and the complex inter-relationship with HIV infection. This may provide many new challenges and open other avenues for dealing with the HIV epidemic and its many facets. Subsequent research needs to be directed to local validation of the SRQ-10. It would also need to assess the mental and behavioral changes occurring in individuals who are HIV positive and are commenced on highly active anti-retroviral drugs. Among the many challenges is how to improve prevention, screening and diagnosis for mental distress as targeted at the most vulnerable groups, such as the poor, the lower educated, the women, the widowed and predominately the HIV infected. Another challenge is strengthening existing mental health facilities and capacity building in order to improve access to universal basic mental health care. This is of critical importance as it would provide knowledge, confer skills necessary for assimilating health promotional information on HIV which in turn is likely to be linked to both reduced risk of mental distress and HIV transmission.

## List of Abbreviations

HIV: Human Immunodeficiency Virus; AIDS: Acquired Immunodeficiency Syndrome; CD4: Cellular differentiation marker 4; HAART: Highly Active Anti-retroviral Therapy; SRQ-10: Self Reporting Questionnaire-10; DSM IV: Diagnostic and Statistical Manual IV; ICD-10: International Classification of Diseases version 10; AMOS 7: Analysis of Moment Structures version 7.0; SEM: Structural Equation Modeling; GFI: Goodness of Fit Index; AGFI: Adjusted Goodness of Fit Index; CFI: Comparative Fit Index; RMSEA: Root Mean Square Error of Approximation.

## Competing interests

The authors declare that they have no competing interests.

## Authors' contributions

PJC and KMF contributed to the analysis and drafting of the manuscript. KMF also contributed to the design, conduct, and critical revision of manuscript and approval of final version.

## Pre-publication history

The pre-publication history for this paper can be accessed here:


